# Obstetric vesico-vaginal fistula with bladder neck loss led to bladder eversion: A rare case report

**DOI:** 10.1016/j.eucr.2026.103429

**Published:** 2026-04-10

**Authors:** Kadek Budi Santosa, Putu Angga Krisna Artha, Parsaoran Nababan, Ronald Sugianto

**Affiliations:** a Department of Urology, Faculty of Medicine, Universitas Udayana, Prof IGNG Ngoerah General Hospital, Denpasar, Indonesia; b Department of Uro-Nephrology, Prof IGNG Ngoerah General Hospital, Denpasar, Indonesia; c Department of Surgery, Faculty of Medicine, Universitas Udayana, Prof IGNG Ngoerah General Hospital, Denpasar, Indonesia; d Department of Urology, Dr. Soedarso General Hospital, Pontianak, Indonesia; e Department of Urology, Faculty of Medicine, Universitas Airlangga, Dr. Soetomo General Academic Hospital, Surabaya, Indonesia

**Keywords:** Bladder outlet obstruction, Bladder eversion, Pelvic organ prolapse, Complication, Reconstruction surgery

## Abstract

Bladder eversion is a very rare urological and gynecological condition characterized by the protrusion of the bladder mucosa through the urethra or vaginal canal. We report a 58-year-old female patient with a bladder eversion caused by an obstetric vesico-vaginal fistula (VVF) with bladder neck loss, along with uterine prolapse grade 2. The surgical technique was a combination of laparoscopy and transvaginal approach, consisting of bladder neck reconstruction, VVF repair, subtotal hysterectomy, and colpo-pectopexy.

## Highlights

• Bladder eversion is a very rare urological and gynecological condition.
• This case presents bladder eversion associated with bladder neck loss and uterine prolapse.
• The management can be performed with a combination of laparoscopy and transvaginal approach.

## 1 Introduction

Bladder eversion is a very rare urological and gynecological condition characterized by the protrusion of the bladder mucosa through the urethra or vaginal canal.^1^^,^^2^ It is most commonly reported in neonates or young children with congenital anomalies, while cases occurring in adults are extremely uncommon. In adults, bladder eversion is usually associated with severe pelvic floor weakness, long-standing pelvic organ prolapse (POP), chronic increased intra-abdominal pressure, or prior pelvic trauma or surgery.^3^ The present case demonstrates an unusual presentation of bladder eversion associated with bladder neck loss and long-standing urinary incontinence. This manuscript was prepared following the CARE guidelines (https://www.care-statement.org).^4^

## 2 Case presentation

A 58-year-old female patient complained of a bulge protruding from the vagina for 15 years, which had been getting worse over the past 20 days. The patient had multipara, with four babies by per vagina. The patient had urinary incontinence for 40 years after the third labor, but the patient was not searching for treatment due to embarrassment and the assumption that the symptoms would be cured spontaneously.

**Fig. 1:**
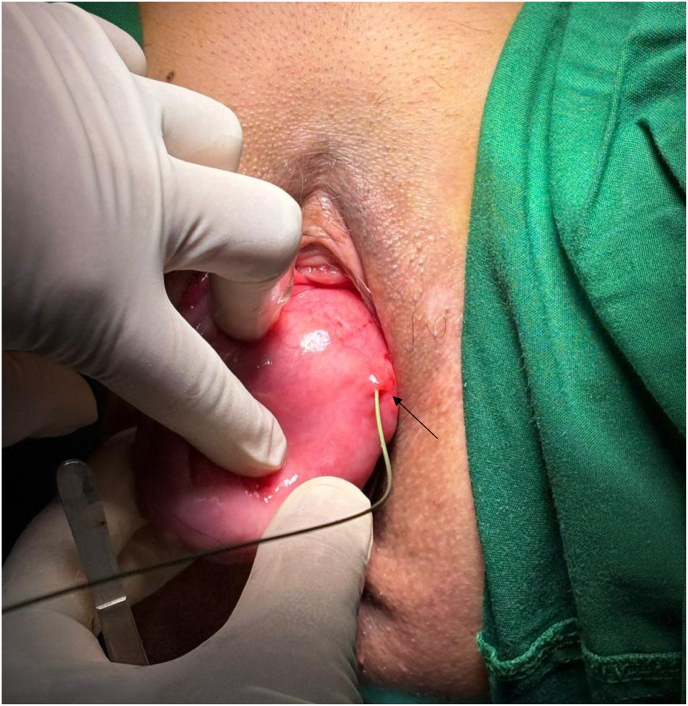
Prolapse of bladder mucosa and ureteral meatal into vagina. The black arrow showed the left ureteral meatus orifice.

On the physical examination, the bulge mass protrudes from the vulva, is mobile, repositionable, soft, and without any tenderness. The prolapsed organ was a bladder eversion with the ureteric orifice into vaginal and a prolapse of the uterus grade II, as shown in Fig. 1.

**Fig. 2:**
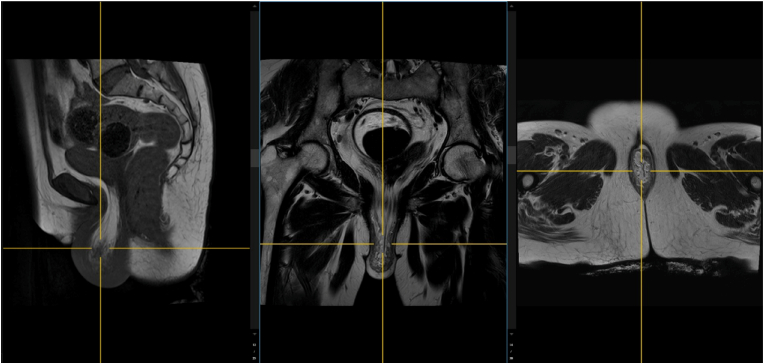
The abdominal MRI with contrast showed the prolapse of the bladder and perivesical fat, moderate uterine descent, mild anorectal junction descent, moderate hiatal enlargement, and severe pelvic floor descent.

The laboratory examination is within normal limits. Then, the abdominal MRI with contrast showed the prolapse of the bladder and peri-vesica fat, moderate uterine descent, mild anorectal junction descent, moderate hiatal enlargement, and severe pelvic floor descent, as shown in Fig. 2. Therefore, the patient was diagnosed with bladder eversion with pelvic organ prolapse and planned for surgical repair.

**Fig. 3:**
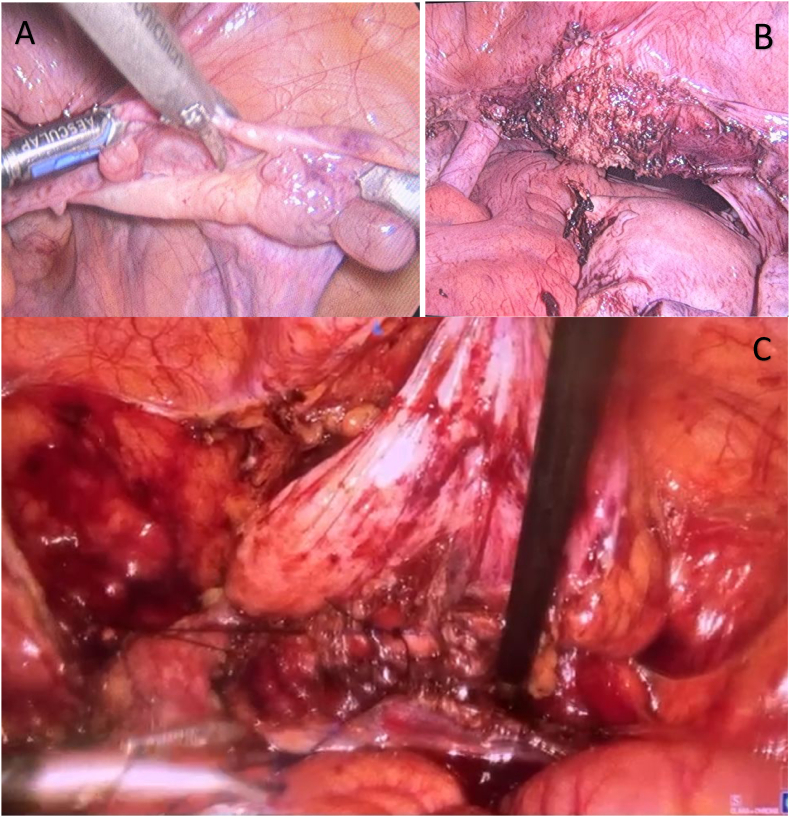
A) Laparoscopy Exploration and ligation of the tuba and round ligament with ovarium preservation. B) Subtotal Hysterectomy. C) Bilateral colpo-pectopexy technique.

**Fig. 4:**
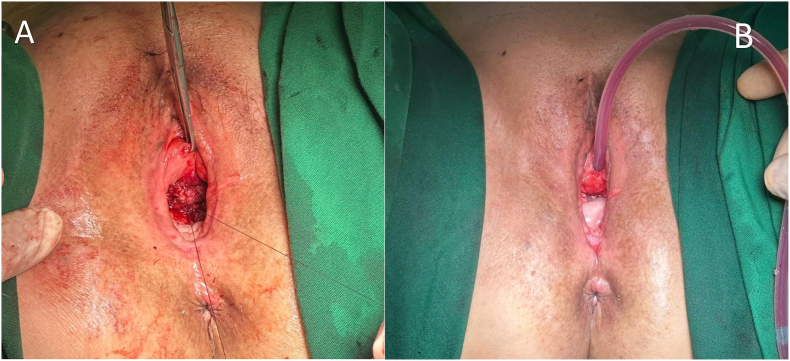
A) The bladder tissue was separated from the vaginal mucosa, and the stenotic bladder neck was excised. B) The reconstructed bladder tissue was anastomosed with the external urethral meatus, while the vagina tissue was reconstructed into a vaginal stump.

**Fig. 5:**
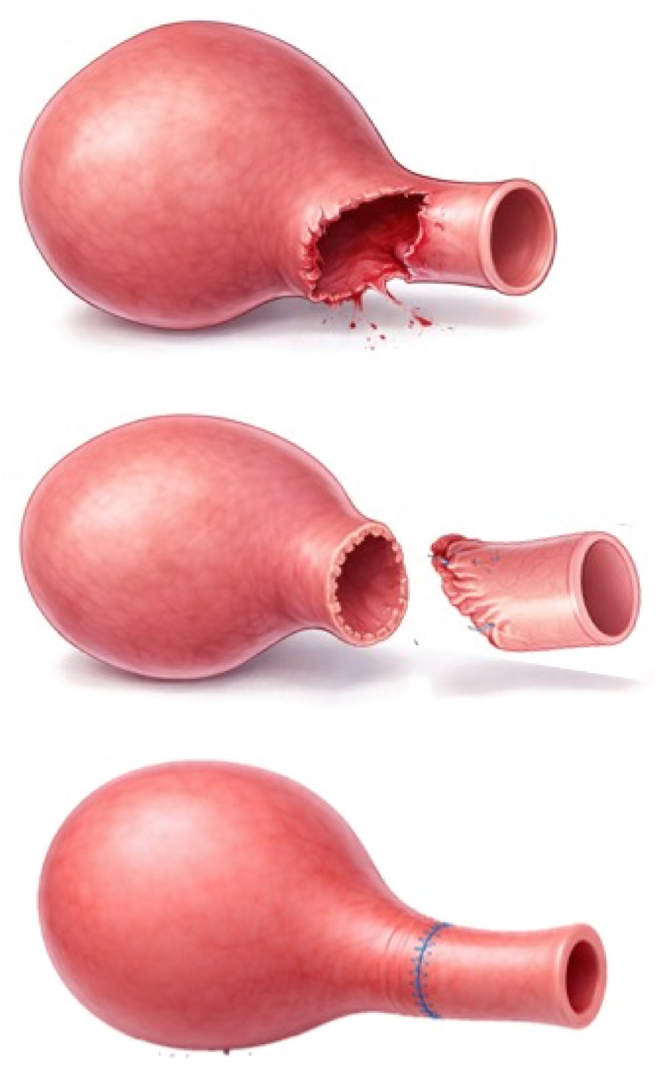
The operative technique involved circumferential excising of the bladder anterior segment and reposition the bladder tissue from the vaginal mucosa. Then, the bladder was reconstructed into the normal bladder anatomy and anastomosed to the external urethral meatus.

The patient was positioned in lithotomy. Subsequently, via a transvaginal approach, an incision was made circumferentially starting from the edge of the fistula, followed by submucosal dissection for 2 to 3 cm to allow for tension-free proximal mobilization of the tissue. During the dissection, fibrosis was noted on the anterior bladder wall. The operation proceeded with a laparoscopic approach; the peritoneum was opened, and the space of Retzius was entered to facilitate dissection of the anterior bladder by incising the endopelvic fascia. During the same laparoscopic session, a subtotal hysterectomy and bilateral colpopectopexy were performed using 1.0 Prolene sutures, as shown in Fig. 3. Subsequently, via the transvaginal approach, the fibrotic tissue at the bladder neck was excised. A dorsal spatulation of the proximal urethra, approximately 1 cm into the normal urethral mucosa, then the bladder ventral segment below pubo-cervical fascia was plicated to equalize the urethra caliber, followed by an end-to-end anastomosis of the dissected bladder segment to the urethra using interrupted 3.0 Vicryl sutures. A 16 Fr silicone catheter was placed. The vaginal mucosa was closed with interrupted 2.0 Vicryl sutures, as shown in Fig. 4. The illustration of the operation technique is shown in Fig. 5.

The patient was planned for vaginal tampon for 24 hours, an indwelling urinary catheter for 8 weeks, and activity limitation to prevent the increasing intra-abdominal pressure. After 8 weeks, the catheter was removed, and the patients could micturate normally, but still had a frequency complaint with stress incontinence. Three months later, the patient was continent with the minimal complaint of frequency.

## 3 Discussion

Pelvic organ prolapse is a common condition in multiparous women, particularly those with a history of vaginal deliveries.^5^ Repeated childbirth can weaken the pelvic floor muscles, connective tissues, and supportive ligaments, predisposing patients to uterine, vaginal, and bladder prolapse.^6^ Our patient had multiple vaginal deliveries and a long history of urinary incontinence following her third labor, suggesting chronic pelvic floor dysfunction. Over time, this pelvic support failure likely contributed to progressive descent of pelvic organs, including the bladder. The persistent high pressure within the bladder combined with weakened pelvic floor support may have facilitated the eversion of the bladder through the vaginal canal.^7^

Previous reports highlight that bladder eversion in adults is commonly associated with severe pelvic organ prolapse or structural defects such as fistulas. These distinctions emphasize the variability in pathophysiology and underscore the importance of individualized surgical management depending on the underlying cause and intraoperative findings.^2^^,^^5^ However, Marantidis et al. reported that primary bladder neck obstruction may lead to structural bladder abnormalities, such as bladder diverticulum, due to chronic outlet obstruction and increased intravesical pressure.^8^ The MRI findings indicate advanced pelvic floor relaxation involving multiple compartments. However, the clinical appearance of bladder eversion in this case was mimicking to other pelvic organ prolapse.

Management of bladder eversion in adults depends on the underlying cause and associated pelvic pathology. The primary goals of treatment include restoring normal bladder anatomy, relieving obstruction, and repairing pelvic floor support. In this case, surgical management involved excision of the stenotic bladder neck, repositioning of the everted bladder tissue, and reconstruction of the bladder anatomy with anastomosis to the external urethral meatus. This approach addressed both the anatomical abnormality and the obstructive component.^9^^,^^10^

Additionally, reconstruction of the vaginal structure and management of pelvic organ prolapse were performed. The separation of the vagina from the uterus followed by subtotal hysterectomy allowed correction of uterine prolapse and reconstruction of the vaginal stump. The use of colpo-pectopexy provided additional support to the bladder and vaginal stump by suspending them to the pectineal ligaments. Compared with sacrocolpopexy, colpo-pectopexy is considered a safe alternative with advantages such as lower risk of bowel complications, preservation of pelvic anatomy, and more feasible in this case.^11^^,^^12^

Postoperative management is also crucial in preventing recurrence and ensuring adequate healing. The use of vaginal packing, prolonged urinary catheterization, and restriction of activities that increase intra-abdominal pressure are important measures to protect the surgical repair.^5^^,^^6^ In this patient, early postoperative follow-up demonstrated good outcomes with no recurrence of prolapse and stable postoperative recovery.

The complaints of urinary frequency and stress urinary incontinence post-operatively may be caused by chronic disuse atrophy, small bladder capacity, and urethral support incompetence.^13^ The strength of this study lies in the presentation of a very rare clinical condition, namely bladder eversion associated with bladder neck loss and uterine prolapse in an adult female, managed by a combined technique that releases the circumferential bladder anterior segment, followed by an end-to-end anastomosis.

## 4 Conclusion

Bladder eversion in adults is a rare condition that can be associated with severe pelvic floor dysfunction and bladder outlet obstruction. Early recognition and comprehensive surgical management addressing both the bladder pathology and pelvic organ prolapse are essential to achieve optimal functional and anatomical outcomes. This case contributes to the limited literature on adult bladder eversion and emphasizes the need for individualized surgical strategies in complex pelvic floor disorders.

## CRediT authorship contribution statement

**Kadek Budi Santosa:** Writing – review & editing, Writing – original draft, Validation, Investigation, Formal analysis, Data curation, Conceptualization. **Putu Angga Krisna Artha:** Writing – review & editing, Writing – original draft, Validation, Investigation, Data curation. **Parsaoran Nababan:** Writing – review & editing, Validation, Supervision, Investigation, Data curation. **Ronald Sugianto:** Writing – review & editing, Validation, Supervision, Investigation, Formal analysis, Data curation.

## Informed consent statement

Informed consent for the publication of this case report and the accompanying images was obtained from the patient's parents in the Indonesian language. The parents acknowledged understand and voluntarily agreed to the publication anonymously. A signed copy of the written consent has been securely stored. This document is available for review upon request by the corresponding author.

## Funding statement

None.

## Conflict of interest

No conflict of interest.
